# Effectiveness of Core-Shell Nanofibers Incorporating Amphotericin B by Solution Blow Spinning Against *Leishmania* and *Candida* Species

**DOI:** 10.3389/fbioe.2020.571821

**Published:** 2020-10-30

**Authors:** Ingrid Morgana Fernandes Gonçalves, Ítalo Martins Rocha, Emanuene Galdino Pires, Isis de Araújo Ferreira Muniz, Panmella Pereira Maciel, Jefferson Muniz de Lima, Iêda Maria Garcia dos Santos, Roberta Bonan Dantas Batista, Eudes Leonnan Gomes de Medeiros, Eliton Souto de Medeiros, Juliano Elvis de Oliveira, Luiz Ricardo Goulart, Paulo Rogério Ferreti Bonan, Lúcio Roberto Cançado Castellano

**Affiliations:** ^1^Human Immunology Research and Education Group (GEPIH), Escola Técnica de Saúde da UFPB, Federal University of Paraíba, João Pessoa, Brazil; ^2^Postgraduate Program in Dentistry (PPGO), Federal University of Paraíba, João Pessoa, Brazil; ^3^Postgraduate Program in Dentistry, Federal University of Pernambuco, Recife, Brazil; ^4^Fuel and Materials Laboratory (LACOM), Federal University of Paraíba, João Pessoa, Brazil; ^5^Postgraduate Program in Materials Engineering, Federal University of Campina Grande, Campina Grande, Brazil; ^6^Postgraduate Program in Materials Engineering, Federal University of Paraíba, João Pessoa, Brazil; ^7^Department of Engineering, Federal University of Lavras, Lavras, Brazil; ^8^Postgraduate Program in Health Sciences, School of Medicine, Federal University of Uberlândia, Uberlândia, Brazil; ^9^Institute of Biochemistry and Genetics, Federal University of Uberlândia, Uberlândia, Brazil; ^10^Department of Medical Microbiology and Immunology, University of California Davis, Davis, CA, United States

**Keywords:** amphotericin B, nanofibers, drug delivery systems, candidiasis, leishmaniasis

## Abstract

The aim of this study was to develop polymeric nanofibers for controlled administration of Amphotericin B (AmpB), using the solution centrifugation technique, characterizing its microstructural and physical properties, release rate, and activity against *Leishmania* and *Candida* species. The core-shell nanofibers incorporated with AmpB were synthesized by Solution Blow Spinning (SBS) and characterized by scanning electron microscopy (SEM), differential scanning calorimetry, X-Ray diffraction, and drug release assay. *In vitro* leishmanicidal and antifungal activity were also evaluated. Fibrous membranes with uniform morphology and smooth surfaces were produced. The intensity of the diffraction peaks becomes slightly more pronounced, assuming the increased crystallization in PLA/PEG at high AmpB loadings. Drug release occurred and the solutions with nanofibers to encourage greater incorporation of AmpB showed a higher concentration. In the results of the experiment with promastigotes, the wells treated with nanofibers containing concentrations of AmpB at 0.25, 0.5, and 1%, did not have any viable cells, similar to the positive control. Various concentrations of AmpB improved the inhibition of fungal growth. The delivery system based on PLA/PEG nanofibers was properly developed for AmpB, presenting a controlled release and a successful encapsulation, as well as antifungal and antileishmanial activity.

## Introduction

In recent years, nanotechnology has sought to study drug delivery systems with great interest. These systems can be characterized by a device capable of being incorporated by therapeutic agents, to control the release and improve the effectiveness of these agents (Chen and Liu, 2015; Tseng and Liu, 2015). The polymeric nanofibers, a nanostructured material, can be used as local delivery systems. They are defined as solid fibers, with a nanoscale diameter, presenting better mechanical properties and flexibility than any other form of the same material (Beachley and Wen, 2010; Bhardwaj and Kundu, 2010).

The solution blow spinning (SBS) is an efficient method to obtain polymeric nanofibers. This technique produces fibers in the same size range as electrospinning, has a greater potential for commercial scale-up, does not use high voltage or an electrically conductive collector and results in nanofibers with high surface area (Medeiros et al., 2009; Oliveira et al., 2011, 2013). The main components of SBS are a concentric spray nozzle and a dispensing system, which controls the amount of polymer delivered to the nozzle. Under the action of compressed air at the outlet of the nozzle, the non-woven material consisting of interlaced polymeric fibers is formed at the collector (Bolbasov et al., 2014). According to their internal structure, they can be classified as uniform and core-shell nanofibers, while according to their orientation they can also be classified as randomly oriented and aligned nanofibers (Pelipenko et al., 2015).

The core-shell nanofibers are moldable structures that allow the encapsulation of bioactive substances and may be able to form viable microenvironments through permeability to exchange nutrients, so that two components can be deposited together forming a continuous core-shell structure. With this, it is possible to add different substances for different applications, such as bioactive glass in an alginate shell and stem cells in the core of collagen, for application in the engineering of bone tissues, allowing cellular proliferation and osteogenic differentiation (Olmos et al., 2015). They also can preserve the molecules encapsulated in the core and can carry and release sequentially growth factors for bone regeneration, such as polyethylene and polycaprolactone core-shell fiber (Kang et al., 2015). In addition to carrying therapeutic substances, these nanoscale systems also have the potential to simulate tissues, with surfaces with therapeutic and adhesion properties and polymers responsible for improving reactivity with cells and a sustained release of drugs (Singh et al., 2015).

The incorporation of drugs in the mesh of these nanofibers offers several benefits, such as high area-surface-volume ratios, high porosity, and the possibility of controlling the crystalline—amorphous phase transition, contributing to a higher dissolution rate and an increase of solubility due to the reduction in particle size. Also, it allows the local delivery of these drugs, and a lower dose can be used and, consequently, a reduction in adverse effects (Blagden et al., 2007; Sebe et al., 2015; Tseng and Liu, 2015). Thus, release control can be achieved using fiber-based formulations and several types of drugs can be incorporated, including antibiotic, antineoplastics, analgesics, non-steroidal anti-inflammatory, antimicrobials and antifungals (Uhrich et al., 1999; Tseng et al., 2013). Among the drugs with this possibility, amphotericin B (AmpB) is a potent anti-leishmanial agent (Sarwar et al., 2018) and an effective antifungal against species of Leishmania and Candida, which acts through its binding to the ergosterol present in the membrane of these pathogens, being able to depolarize it, increasing permeability, resulting in an osmotic imbalance with consequent cell death (Quindós et al., 2019).

Therefore, this drug is very useful in the treatment of cases of candidosis, cryptococcosis, histoplasmosis, blastomycosis, paracoccidioidomycosis, coccidioidomycosis, aspergillosis, extracutaneous sporotrichosis, mucormycosis, hyalohyphomycosis, phaeohyphomycosis, and leishmaniasis (Ellis, 2002; Mattos et al., 2015; Turner et al., 2015). For granulomatous and invasive forms of candidosis, AmpB and other antifungals such triazoles, allylamines (terbinafine), and echinocandins (caspofungin), are elective drugs with good clinical outcomes (López-Martínez, 2010). However, unwanted characteristics of AmpB, including high cost, toxicity, and necessity of parenteral administration, may limit its use (Mattos et al., 2015; Turner et al., 2015; Kariyawasam et al., 2019). For this reason, other vehicles have been developed with the aim of developing new strategies for the administration of this drug, such as films, nanofibers, nanoformulations, as well as systems focused on delivery to the oral mucosa (Hodiamont et al., 2015; Sarwar et al., 2018; Souza et al., 2018; Edmans et al., 2020).

Among these vehicles, nanocomposites are drug delivery systems, whose formulation with different associated polymers may be able to optimize the properties of the controlled release of drugs, with different applications, among them, films for wound healing (Hasan et al., 2017). Likewise, nanoemulsions are also used as a way to reduce the side effects of some drugs, such as antineoplastic drugs, which when administered in this way allow hydrophobic molecules to be transported followed by a controlled release in the target cells (Fopase et al., 2020). Or even nanoemulsions, which could be used to protect bioactive substances from degradation, optimizing the bioavailability of compounds, presenting antimicrobial activity and biocompatibility, such as food supplements consisting of coconut oil and tocopherol (Saxena et al., 2017).

In this sense, it would be possible to direct the use of polymeric nanocarriers with AmpB for the treatment of two diseases that have quite significant oral repercussions of interest to dentistry, candidiasis and leishmaniasis. Oral candidiasis is a fungal infection caused by the genus *Candida* and can be presented in different clinical forms, such as pseudomembranous, erythematous, atrophic, angular cheilitis (Hellstein and Marek, 2019). Leishmaniasis is a disease caused by protozoa of the genus *Leishmania*, which can be visceral, cutaneous, and mucocutaneous, which can present lesions with impairment of the oral mucosa (Almeida et al., 2016). Thus, the use of polymeric delivery vehicles could represent a new perspective in the treatment of these diseases. So, the objective of this study was to develop polymeric nanofibers for controlled administration of Amphotericin B, using the solution centrifugation technique, characterizing its microstructural and physical properties, release rate, and activity against *Leishmania* and *Candida* species.

## Materials and Methods

### Materials

The polymers utilized were poly (L-lactic acid) (PLLA) (Mw = 1.25 × 10^5^ g/mol) and poly (ethylene glycol) (PEG) (Mw = 0.4 × 10^4^ g/mol), both purchased by Perstorp (Warringtonn, United Kingdom) in pellet form. The solvents used are chloroform (CHCl_3_, 99%), acetone [(CH_3_)_2_CO, 99.5%], and glacial acetic acid (C_2_H_4_O_2_), manufactured by F. Maia (Brazil). The drug used is Amphotericin B (AmpB) (Sigma–Aldrich).

### Synthesis of Core-Shell Nanofibers Incorporating AmpB by Solution Blow Spinning

Initially, polymer solutions were prepared by solubilizing PLA (10%, m/v) in chloroform:acetone (3:1 v/v). PEG was added at 10% (w/w) in relation to PLA. The AmpB was diluted in 10 mL of acetic acid, at 0.25, 0.5 e 1% (w/w) in relation to the total mass of PLA/PEG. In the formation of core-shell morphology two different materials are delivered independently through a co-axial capillary and drawn to generate nanofibers in core-sheath configuration (Elahi et al., 2013). For the SBS technique the system structure is composed by three concentric tubes in which a polymer blend solution (PLA/PEG), AmpB solution and pressurized air overpass separately. These elements only come into contact at the outlet of the tubes. A metallic collector, under rotation of 550 RPM, was positioned to allow 200 mm of protruding tip from the inner tube. The fibers produced were deposited on a collector, previously coated with aluminum (Oliveira et al., 2013; Bonan et al., 2015). The injection rate of the polymer solution was 8.4 mL/h on the outer tube, the rate of 7.5 mL/h was applied to drug solution in the inner tube and the pressure for the air was 30 Psi.

### Characterization of Core Shell Nanofibers Incorporating Amphotericin B by Solution Blow Spinning

#### Scanning Electron Microscopy (SEM)

The morphology of the fibers was observed using a scanning electron microscope (SEM) model Zeiss LEO 1430. The samples were collected in aluminum foil, cut with a blade, fixed on aluminum stubs used double-sized adhesive tape and their surfaces covered with gold K 550X Emitech sputter coater. The diameters of the fibers were evaluated by image analyzer software (Image J, National Institutes of Health, United States). For each sample, the average diameter and its dispersion were determined from the analysis of 80 randomly selected fibers.

#### X-Ray Diffraction

The models for diffraction of the samples were obtained from a SHIMADZU XRD – 6000 diffractometer. The fibers were spun onto aluminum sheets and subsequently cut into 25×25 mm sizes. Sweeps were conducted from 4 to 40° (2θ) at a speed of 0,01° per 0,5 seconds. The copper alpha radiation (CuK, λ = 0.154 nm) filtered with nickel filter was employed in the analyses.

#### Drug Release Assay

The immersion method was used to study the release characteristics of AmpB from the nanofibers. Ultraviolet visible spectra were recorded using UV-Vis equipment (spectroscopy in the range of UV/VIS, Perkin Elmer equipment, Lambda 25) for the calibration curve preparation and monitoring the drug release. Scans were performed at 380 nm wavelength with pre-determined AmpB concentrations of 0.5, 0.25, 0.12, 0.06, 0.03, and 0.015 mg/mL to determine the calibration curve. After this determination, samples of 2×2 cm^2^ of each fiber were immersed in 3 mL of phosphate buffer (PBS) with pH 7.4. After the predetermined time intervals (60 min, 24 h, 7 days), the total volume of the solution was removed and analyzed in the UV-VIS equipment in length wave of 380 nm. Then, the solution was again brought into contact with the nanofibers until the next evaluation. The reference used for comparison was PBS. The release during the study was calculated by comparing the release profile with the standard calibration curve (Nanda and Mishra, 2011).

### *In vitro* Leishmanicidal Activity

#### Cultivation of Leishmania

*Leishmania amazonensis* and *Leishmania braziliensis* were routinely maintained as promastigotes in RPMI 1640 medium (Gibco) at 26°C supplemented with heat inactivated (30 min, 56°C) fetal bovine serum (FBS) (Sera Laboratories International, Horsted Keynes, United Kingdom) and 100 U/mL penicillin + 100 μg/mL streptomycin (BioWhittaker, Verviers, Belgium) in 25 mL culture flasks.

#### Promastigote Assay With Absorbance Reading

The experiment with promastigotes were made in flat-bottomed 96-well cell culture microtiter plates with lids (Falcon II, BD, Bedford, MA, United States), using one plate for absorbance. Cultures were carried out at 26°C in an aerated culture chamber or incubated in a 95% air/5% CO_2_ humidified atmosphere. Culture media (RPMI 1640) with additional 20 mM Hepes was used. 2.5 × 10^5^ per well of mid-log phase promastigotes of *L.amazonensis* and *L. braziliensis* were added, with a culture media, in a final volume of 190 μL/well in 96-microtiter plates. Ten microliters of AmphB at a concentration of 10 mM were used as controls and PLA/PEG without drugs was also inserted. Sterile control was obtained after 4 cycles of 480 min on ultrasound appliance. Cultures were carried out at 26°C in plates with lids under a 5% CO_2_ atmosphere. After 24 h incubation, 20 μL of 0.5 mM Resazurin was added and the plates were kept for 136 h. The readings of absorbance were obtained using a dual filter of 560 and 600 nm. After 136 h of initial inoculation, a count was made using Neubauer chamber to estimate the number of viable cells per well (Corral et al., 2013).

### *In vitro* Antifungal Activity

#### Strains of *Candida* and Preparation of Inoculum

The strains of *Candida albicans* (ATCC 10221), *Candida glabrata* (ATCC 2001) *Candida krusei* (ATCC 34135), and *Candida tropicalis* (ATCC 750) were used in this study. These were cultured in Sabouraud Dextrose Broth—SDB (Acumedia^®^, Neogen – Lesher Place Lansing, Michigan 48912, United States) and incubated at 37°C for 48 h. Suspensions of these microorganisms were prepared in saline solution (0.9% NaCl) and adjusted to 1 × 10^6^ CFU/mL.

#### Assay to Evaluate the Antifungal Activity of AmpB

The agar diffusion method was used to evaluate the antifungal agent. For this, 20 mL of Sabouraud Dextrose Agar (SDA) (HIMEDIA^®^, Place Laboratories. Paraná, Brazil), was melted and cooled to 45–50°C and dispensed into sterile Petri dishes (KASVI^®^, Paraná, Brazil). With the solidification of the agar, 1mL of fungal suspension at a concentration of 1 × 10^6^ CFU/mL was inoculated on each plate. Sterilized filter disks with 55 μl of 10 mM AmpB were used as positive controls and sterilized filter disks with 55 μl of saline solution as negative controls. The disks were placed, and the cultivation was made in 37°C for 48 H into a bacteriological incubator. After the cultivation, the diameters of inhibition halos were measured. All measures were made by triplicate. The measurement of the diameters of inhibition zones of fungal growth was performed using the software ImageJ (Su et al., 2012).

#### Disk Diffusion Assay

For direct contact/plating assay, assay tubes with 1 mL Sabouraud dextrose Broth (HIMEDIA^®^, Place Laboratories. Paraná, Brazil) were incubated with reference strains and control/test disks for 2 h, under shaking, at 37°C. after 10 microliters of this solution plating into SDA in increased dilutions (10^–1^ to 10^–4^). After incubation for 48 h at 37°C, CFU were counted and the final values were expressed in CFU/mL, according to the NCCLS Guideline (M44-A), modified (Rolón et al., 2006).

## Results

### Morphology of Nanofibers Mats

**FIGURE 1:**
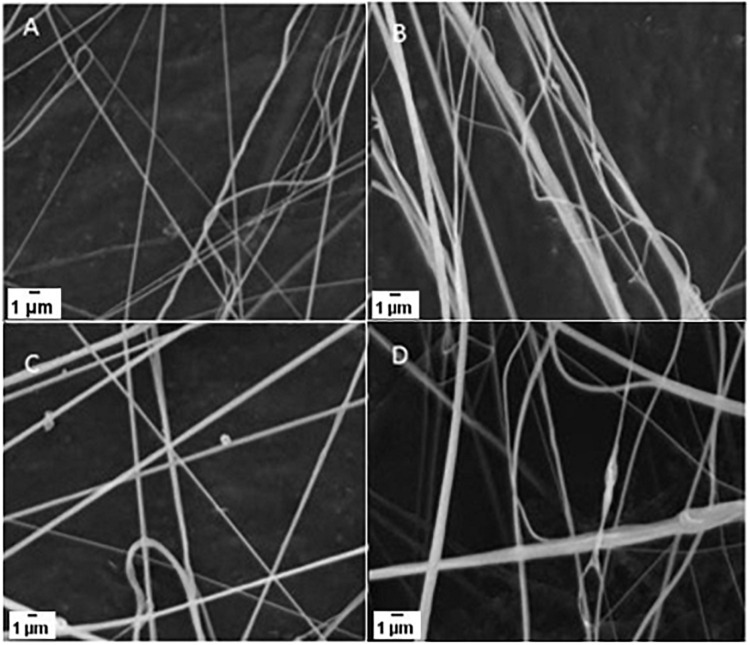
MEV photomicrographs showing PLA/PEG NF **(A)** and PLA/PEG NF with 0.25% of AmpB **(B)**, PLA/PEG NF with 0.5% of AmpB **(C)** and PLA/PEG NF with 1% of AmpB **(D)**.

The Figure 1 shows the morphology of the core-shell nanofibers. Fibrous membranes with uniform morphology and smooth surfaces were produced. The results of fiber diameter analyses show that the addition of AmpB led to an increase in average fiber diameter as compared to PLA/PEG nanofibers non- charged with the drug (Table 1). Groups containing drugs showed no statistical difference among them, regardless of the quantity of AmpB incorporated.

**TABLE 1:** Average diameter and diameter dispersion of nanofibers.

Sample	Averagediameter(nanometers)
PLA/PEG	92.2222.09^a^
PLA/PEG/AMP 0.25%	119.8330.28^b^
PLA/PEG/AMP 0.5%	115.0725.10^b^
PLA/PEG/AMP 1%	110.2729.06^b^

*Identical superscripted small letters denote no significant differences among groups (p > 0.05) (Kruskal–Wallis test).*

### Crystallographic Characterization

**FIGURE 2:**
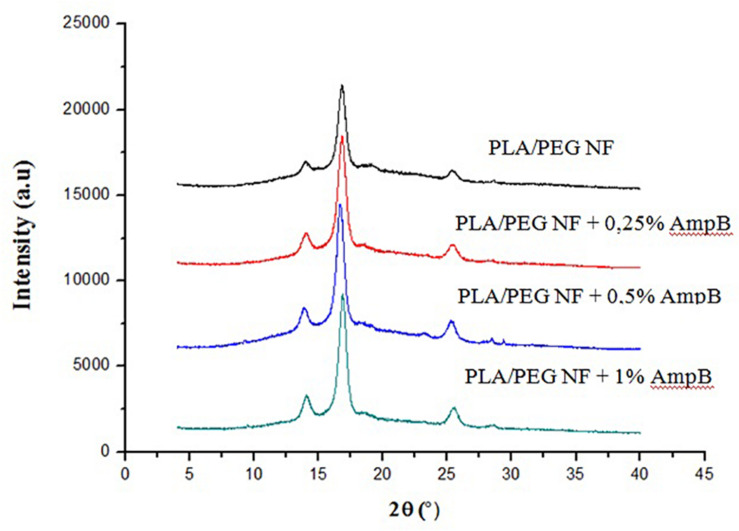
X-ray diffraction patterns of PLA-PEG and PLA–PEG-AmpB nanofibers.

Figure 2 shows the X-Ray diffraction patterns of the evaluated samples. All samples show similar diffraction patterns, presenting two reflection peaks (near 13 and 16°), ascribed to α crystals, and a small peak (near 25°) associated with the β phase of poly (lactic acid). As the Amphotericin B (AmpB) loading is increased, the intensity of the diffraction peaks becomes slightly more pronounced, assuming the increased crystallization in PLA/PEG at high AmpB loadings.

### *In vitro* Controlled Release of AmpB

**FIGURE 3:**
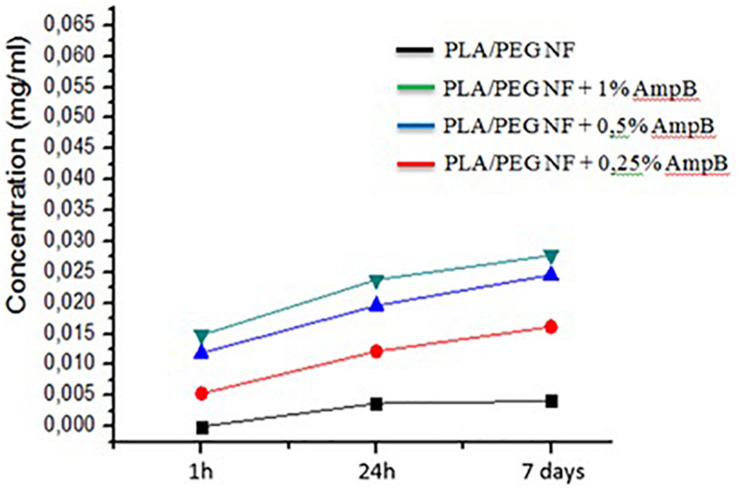
Concentration of amphotericin B (mg/mL) in the solution of PBS.

Figure 3 shows the concentration (mg/mL) of amphotericin B based on absorbance reading performed by UV-Vis equipment (spectroscopy in the range of UV/VIS, Perkin Elmer equipment, Lambda 25). The drug concentration in the solution was calculated from the equation (Y = 3.7739 X + 0.0035) obtained from the calibration curve, in which Y is absorbance and X is concentration. The value of *R*^2^ in the curve was 0.9982. The results showed that, in an evaluation of 1 h, drug release happened and the solutions with nanofibers to encourage greater incorporation of AmpB showed a higher concentration. In the following readings (24 h and 7 days), the concentration of the solutions showed a gradual increase (Hasan et al., 2017).

### *In vitro* Antileishmanial and Antifungal Activity

**FIGURE 4:**
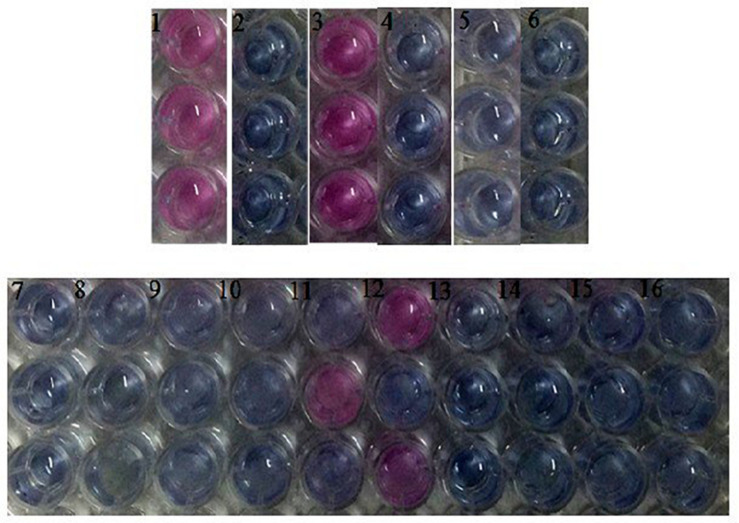
Microplate after 136 H of inoculation with test groups placed on triplicate. (1) Growth control (*L.amazonensis)*; (2) 10 mM AmpB; (3) Growth control (*L. braziliensis*); (4) Negative control (RPMI); (5) Sterile control^1^; (6) Sterile control^2^; (7) Positive control [10 mM AmpB]^1^; (8) PLA/PEG nanofiber with 1% AmpB^1^; (9) PLA/PEG nanofiber with 0.5% AmpB^1^; (10) PLA/PEG nanofiber with 0.25% Amp B^1^; (11) PLA/PEG nanofiber^1^; (12) PLA/PEG nanofiber^2^; (13) Positive control [10 Mm AmpB]^2^; (14) PLA/PEG nanofiber with 1% Amp B^2^; (15) PLA/PEG nanofiber with 0.5% AmpB^2^; (16) PLA/PEG nanofiber with 0.25% AmpB^2^.^1^Against to *L.amazonensis*, ^2^Against to *L. braziliensis.*

Figure 4 shows the visual results of the experiment with promastigotes, using one plate for absorbance. Resazurin dye (7-Hydroxy-3H-phenoxazin-3-one 10-oxide) was employed for promastigote viability testing. Resazurin is a redox potential indicator that is converted to fluorescent and colorimetric resorufin dye by the metabolically active cells. After 136 h of inoculation with test groups, it was found that the cells treated with nanofibers loading AmpB in concentrations of 1, 0.5, and 0.25% (collums 8–10 and 14–16) did not present the pinkish characteristic of resofurin.

**FIGURE 5:**
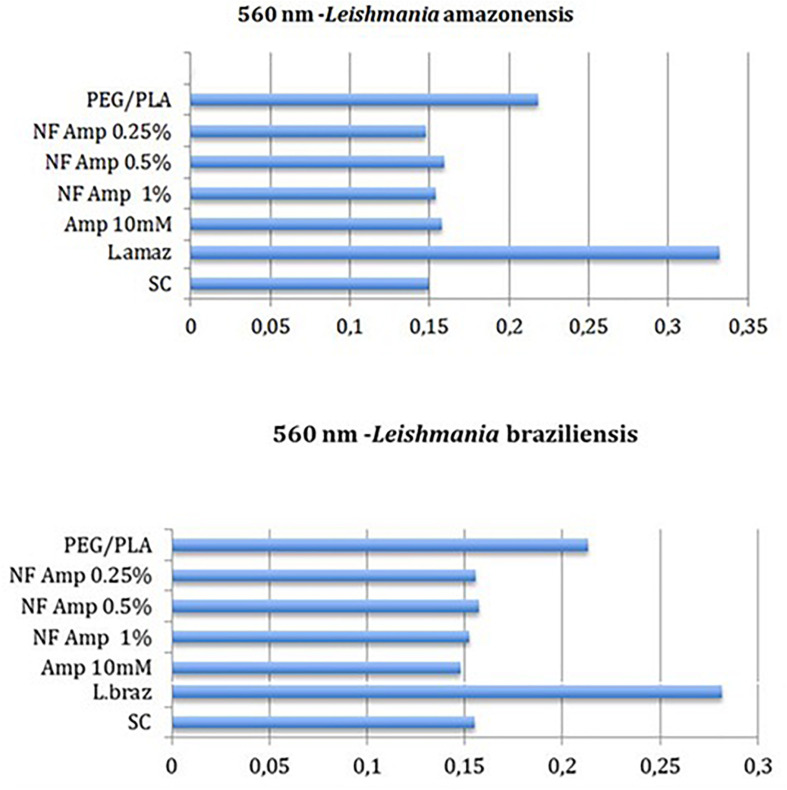
Absorbance reading focusing nanofibers, free drugs, controls and species of *Leishmania* (560 nm filter, 136 H). SC, Sterile Control; NF, Nanofibers.

Concentration of resofurin, the product of reduction of resazurin, in the *Leishmania* cultures was determined by reading the absorbance. The results of reading of absorbance (Figure 5) shows that the absorbance decreased when the nanofibers were utilized compared to the growth control. Resazurin was effectively reduced to resorufin in the growth control, evidenced by the higher levels of absorbance. After the reading of absorbance, an aliquot of 10 microliters from each well was removed and a count was made using Neubauer chamber to estimate the number of viable cells per well. The Table 2 shows that in the wells treated with nanofibers containing concentration of AmpB at 0.25, 0.5, and 1%, did not have any viable cells, similar to the positive control. The nanofiber free of AmpB presented a count of viable cell smaller than growth control. In Table 3, the percentage reduction of 0.5mM Resazurin according to *Leishmania* species is exposed.

**TABLE 2:** Viable cell count per well according to *Leishmania* species and evaluated groups.

Group	*L. amazonensis*	*L. braziliensis*
Growth Control	5.6 × 10^7^	1.78 × 10^7^
PLA/PEG	1.93 × 10^7^	2.28 × 10^6^
10 mM AmpB	n.d.	n.d
PLA/PEG NF with 1% AmpB	n.d.	n.d
PLA/PEG NF with 1% Amp B	n.d.	n.d
PLA/PEG NF with 0.5% AmpB	n.d.	n.d
PLA/PEG NF with 0.25% Amph B	n.d.	n.d

*n.d, non-detected.*

**TABLE 3:** Percentage reduction of 0.5 mM Resazurin according to *Leishmania* species (136 H).

Species	10 mM AmpB	PLA/PEG nanofiber with 1% AmpB	PLA/PEG nanofiber with 0.5% AmpB	PLA/PEG nanofiber with 0.25% Amph B	PLA/PEG
*L. amazonensis*	3.8	10.0	10.6	9.3	52.6
*L. braziliensis*	5.4	8.0	8.5	8.0	52.2

**FIGURE 6:**
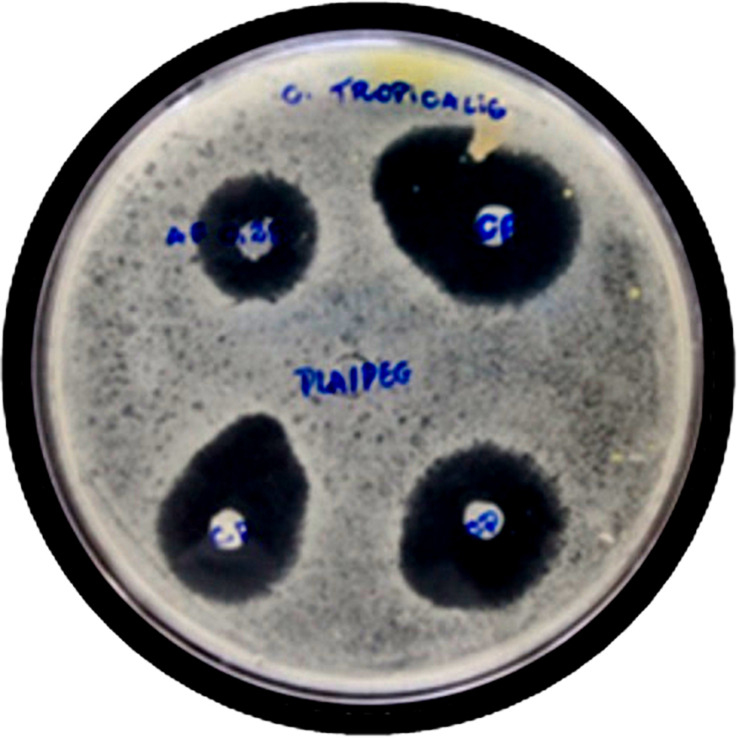
Illustration of inhibition halos formed in the inhibition of fungal growth, *C. tropicalis*, A – NF PLA10/PEG10 0.25% AmpB, B – CP-Positive control and C – Negative Control- PLA-PEG + Acetic acid.

Various concentrations of AmpB improved the inhibition of fungal growth. The drug acts likely increase in membrane permeability, resulting in the formation of ion channels, which can be the cause of oxidative damage of the constituents of the membrane, demonstrating their antifungal activity (Kim et al., 2003; Spasova et al., 2007). The results indicate that the polymer matrix allows the release of the drug through the PEG. The Table 4 shows that the inhibition halo formed by concentration of 0.25% AmpB was similar to the highest concentration of 1% AmpB, according to the species of *Candida* in comparison to the positive control (PC). These data suggest that with this type of technology to have an effective inhibition zone it is only necessary to have small concentrations of the drug, which varies according to the species of *Candida*. To corroborate this finding, it was observed that PEG, to have a hydrophilic and hygroscopic nature, can be transferred to a greater interaction with the microbial means and in turn degrade and dissolve PLA fiber, resulting in higher release rates that form halos inhibition. *Candida albicans* 1% AmpB among groups and CP showed a significance level of *p* < 0.033. The other groups have shown no statistical significance compared to the CP with *p* > 0.05, illustrated by the Figure 6. The Table 5 shows the antimicrobial activity of PLA/PEG nanofibers against multiple species of *Candida* (CFU/mL), in which 0 colony of *C. glabrata* at 0.25% concentration was observed, while *C. albicans* had an increase at the same concentration. This demonstrates that variations in drug concentrations promoted different results in the number of colonies, in a species-specific manner.

**TABLE 4:** Median halos disk diffusion testing of *Candida* species in millimeters.

Species	PC	NC	PLA10/PEG10	PLA10/PEG10 1%AmpB	PLA10/PEG100.5% AmpB	PLA10/PEG10 0.25% AmpB
*C. albicans*	31.36 (1.79)	0	0	15.53 (1.59)	25.14 (1.44)	19.29 (1.58)
*C. glabrata*	32.62 (3.48)	0	0	22.81 (0.26)	21.72 (2.16)	21.82 (3.67)
*C. krusei*	19.57 (1.49)	0	0	17.90 (0.41)	18.00 (2.14)	18.34 (0.09)
*C. tropicalis*	27.47 (0.58)	0	0	20.52 (3.26)	20.3 (1.27)	20.51 (0.03)

**TABLE 5:** Antimicrobial activity of PLA/PEG nanofibers against multiple species of *Candida* (CFU/mL).

Species	PC	NC	PLA10/PEG10	PLA10/PEG10 1% AmpB	PLA10/PEG10 0.5% AmpB	PLA10/PEG10 0.25% AmpB
*C. albicans*	3 × 10^5^	7 × 10^6^	3 × 10^7^	1.2 × 10^6^	1.6 × 10^6^	3 × 10^7^
*C. glabrata*	3 × 10^6^	1.3 × 10^7^	1.6 × 10^7^	8 × 10^6^	5 × 10^6^	0
*C. krusei*	1.3 × 10^6^	2 × 10^7^	2 × 10^7^	1.1 × 10^7^	1.2 × 10^7^	1.0 × 10^7^
*C. tropicalis*	0	2.2 × 10^6^	2 × 10^6^	1.2 × 10^6^	1.1 × 10^6^	2 × 10^6^

## Discussion

Different fiber morphologies can be obtained according to changes of electrospinning parameters, generating several applications, from tissue engineering to local drug delivery systems. The high surface area, porous structure, possible adjustments in composition, have made the use of polymeric nanofibers as a drug delivery system an attractive alternative (Tungprapa et al., 2007; Kadavil et al., 2019). The use of poly (lactic acid) in this study is justified by the fact that it is biodegradable, compostable, biocompatible and non-toxic, although it has some limitations, such as low rate of degradation concerning other polymers and hydrophobicity (Kim et al., 2003; Silverajah et al., 2012). The incorporation of a hydrophilic polymer, such as poly (ethylene glycol), is an easily achievable approach to modulate the hydrophilicity of the nanofiber (Spasova et al., 2007).

The average diameters ranged from 92 nm for PLA/PEG fibers to about 119 nm for fibers with AmpB. The increase in the average fiber diameter due to the addition of active agents is similar to the results found in the literature (Zeng et al., 2005; Oliveira et al., 2013) and can be interpreted as a success in the incorporation of amphotericin in the PLA/PEG matrix. Three factors contribute to the final fiber morphology: solution concentration, pressure, and injection rate used (Oliveira et al., 2011). During spinning, for all samples, the rates of injection and pressure were kept constant. Although the solution concentration of the core has changed, the amount of amphotericin B incorporated not influence the diameter of the fiber. On the other hand, a study that developed electrospun fiber scaffolds with PLGA [poly (lactide-co-glycolide)] and PEG incorporated with acyclovir, through different proportions of PEG obtained different morphologies, in which this variation resulted in thicker fibers (Wang and Windbergs, 2018).

The variability of the average diameters of the fibers in the SEM can be an important factor to be considered in the release and the nanostructure, and these variations are characteristic of the technique adopted. The construction in the concentric nozzles model promoting the spinning of the blankets in the core-shell model presents a better distribution of formation throughout the blankets. This should be better clarified by evaluating future blending formation projects in different formulations to better understand the process. The great advantage of this system is that there is no chemical interactivity, such as electric charges, which could modify the composition of the drug, altering its biological action, in addition to not using an electric field to stretch the fiber (Medeiros et al., 2009).

The interactions between drug and polymer on a nanostructured system can promote greater difficulty in dissolving AmpB, and just as the physical formulation of the state, crystalline or amorphous, also play an important role in controlling the release of the drug (Essa et al., 2010; Freire et al., 2017). Amphotericin B has a crystalline structure, which contributes to their poor solubility (Senna and Nakayama, 2009). For this reason, the intensity of the diffraction peaks becomes slightly more pronounced, when amphotericin was incorporated into the nanofibers. The reflection peaks (near 13 and 16°) are assigned to α crystals and the small peak (near 24.8°) is associated with the β phase of poly (lactic acid). The formation of β crystals is caused by the different extent of deformation of the polymer molecules during fiber formation by SBS (Hoogsteen et al., 1990; Oliveira et al., 2012). In general, nanofibers tend to be more amorphous, due to the speed of their formation in the electrospinning process (Nematpoura et al., 2020).

The release of amphotericin B was studied at 37°C in phosphate buffer (3 mL) at pH 7.4. The concentration of AmphB in the solution shows that some release occurs in the first hour of immersion (Figure 3). The concentration of the solution was found to vary as a function of drug concentration on the nanofiber, where more concentrated solutions were contacted fibers with higher concentrations of Amphotericin B. The slow and gradual increase in the concentration of AmpB can be attributed to the structure of core-shell, because when the drug is encapsulated in the core of the nanofibers, the release profile presents a slow and steadily increasing profile. The mechanism of release of drugs is affected by the diffusion and degradation of the polymer. Initially, the drugs were released from the fibrous by diffusion through the pores of the fibers and after this, changed to a combination with diffusion and erosion mechanism (Su et al., 2012).

The presence of a hydrophilic polymer (PEG) at the blend may have contributed to the effectiveness of the nanofiber, because it allows the release of the drug. That is, the release kinetics of the drug may be dependent on the proportion of PEG in the sample, since Wang et al. obtained as a result of a slow release in the addition of 5% PEG and increased in the release rate with a 10%, suggesting that it is not only the diameter affecting the surface/volume ratio but also the dissolution capacity of the polymer influences the release of the drug (Wang and Windbergs, 2018). Similar to what occurred in another study, the addition of polyvinylpyrrolidone (PVP), a polymer which is also hydrophilic, in the blend of poly (lactic acid) (PLA) and Copaíba increased both the diameter and the antimicrobial activity of the nanofiber, indicating that the PVP allowed the release of copaiba oil (Bonan et al., 2015).

The results shown are encouraging and corroborate the results found in the literature (Kim et al., 2004; Tort et al., 2019). Different polymeric systems were evaluated, including poly (vinyl alcohol) (PVA) and AmpB fibers, which obtained the greatest release of the drug to which the hydrophilic nature of the polymer was attributed, as this favors the entry of water in the matrix and provides the solvation of AmpB, therefore there is an association between wettability and release kinetics. In the analysis of PLA with AmpB, they found the lowest release, and attributed it to the hydrophobicity of this polymer, as well as to greater interaction of PLA to AmpB, interfering in the dissolution of the drug. Thus, the determination of the speed of release becomes relevant due to its direct influence on the plasma concentrations of the drug (Freire et al., 2017).

The screening of new substances has mainly involved the microscopic count, a laborious process that requires time and is highly dependent on the observer. Colorimetric methods have been reported and demonstrate greater accuracy in relation to the microscopic counting (Rolón et al., 2006). The tests with Leishmania were conducted using resazurin colorimetric methods, in view of its advantages, like simplicity of use, rapidity, reliability, low cost, absence of toxicity and can be used with long incubation periods (Borra et al., 2009; Corral et al., 2013), because promastigote multiplication and inhibition need at least 72 h of observation for significant absorbance variations (Corral et al., 2013). After 136 h of inoculation, the absence of pinkish characteristics of resofurin in the treated groups had shown that the cells treated with nanofibers loaded with AmpB may have lost their capacity to reduce resazurin. A decrease in absorbance values in the groups in contact with nanofibers loaded with AmpB compared to growth control values was found and can be attributed to the reduction in cell viability, because non-viable cells rapidly lose their metabolic capacity to reduce resazurin in the mitochondrion and, thus, do not produce fluorescent signals anymore (Marinho et al., 2014).

The percentage reduction of resazurin correlates with the number of live organisms, such as bacterial, fungi and mammalian cells (Borra et al., 2009). The absence of viable cells in contact with the AmpB nanofibers confirms that, when the percentage reduction in resazurin is low, the cells were infeasible. The effectiveness of nanofiber to inhibit the growth and proliferation of Leishmania was confirmed and it proved to be as effective as the free drug. The nanofibers characteristic of having slow and gradual release of the drug also supports the claim that the developed nanofibers are as effective in inhibiting the growth of *Leishmani*a, as the free drug or a standard commercial formulation. Similar results were found in the literature for Candida species (Lakshminarayanan et al., 2014).

The *in vitro* experiments with anticandidal and antileishmanial action of drugs followed the reference protocols, in which they use 10mM AmpB as a standard for antimicrobial action (Su et al., 2012; Corral et al., 2013). The results obtained in the present study with concentrations 4 times lower, released over time in a controlled release system of PLA/PEG proved to be as better as the control. Thus, it is demonstrated that the nanoscale delivery system for the treatment of protozoa and fungi using lower concentrations of the drug, reduces the dose, toxicity, promoting safety in administration, tolerability, in addition to having positive impacts to avoid selection of resistant pathogens.

Amphotericin B-Loaded poly(lactic-co-glycolic acid) (PLGA) nanofibers were developed for application in vulvovaginal candidiasis and tested against reference strains of *Candida albicans*, *Candida krusei*, *Candida glabrata*, and *Candida tropicalis*, using agar diffusion test. The released drug inhibited the growth of these strains although the diameter of the inhibition zone was smaller compared to pure AmpB. However, for this test, all AmpB was available through all the period evaluated, unlike nanofibers in which the release occurred in smaller quantities at the same time, which was the factor that did not allow the formation of larger inhibition zone. Also, under dynamic conditions these nanofibers would promote a constant release, for a longer period favoring the inhibition of microrganisms, presenting itself as an effective measure against disease recurrence (Souza et al., 2018). Thus, nanofibers can act as a potential drug delivery system to model drug AmpB, and could be promising for the treatment of leishmaniasis and severe fungal infections (Nanda and Mishra, 2011).

According to the literature, PLA/PEG 70/30 fibers have thermal stability (Chen et al., 2015). In the present study, the nanofibers were stored at room temperature and did not undergo a dissolution or degradation process, remaining viable for use in the human body. As well as cytocompatibility, which, although it was not performed in this study, is also reported in the literature (Bakandritsos et al., 2010). In this sense, the objective of producing PLA/PEG fibers was to focus on a final product with longer degradation time and storage at room temperature, which could be commercialized and distributed under environmental conditions to endemic areas.

The absence of *in vivo* tests and cytotoxicity assays for cells of the human immune system are limitations of the present study. However, further studies should be carried out to address the existing gaps and to evaluate the therapeutic efficacy of this new biomaterial for future clinical applications in patients affected by these diseases.

## Conclusion

In summary, the delivery system based on PLA/PEG nanofiber was properly developed for AmpB, presenting a controlled release and a successful encapsulation, as well as antifungal and antileishmanial activity. Thus, the technique should be considered an option in the manufacture of drug delivery systems as a therapeutic alternative for the treatment of fungal diseases and leishmaniasis.

## Data Availability Statement

The raw data supporting the conclusions of this article will be made available by the authors, without undue reservation.

## Author Contributions

IG, ÍR, EP, RB, LG, PB, LC worked on conception, designing, experimentation, analyzing and interpretation the data and writing of the manuscript. IM, PM, JML, IS, ELM, ESM, JdO participated of the experimentation, analysis and writing of the article. All authors contributed to the article and approved the submitted version.

## Conflict of Interest

The authors declare that the research was conducted in the absence of any commercial or financial relationships that could be construed as a potential conflict of interest.
